# Large-scale sequence analysis reveals novel human-adaptive markers in PB2 segment of seasonal influenza A viruses

**DOI:** 10.1038/s41426-018-0050-0

**Published:** 2018-03-29

**Authors:** Lei Wen, Hin Chu, Bosco Ho-Yin Wong, Dong Wang, Cun Li, Xiaoyu Zhao, Man-Chun Chiu, Shuofeng Yuan, Yanhui Fan, Honglin Chen, Jie Zhou, Kwok-Yung Yuen

**Affiliations:** 1 Department of Microbiology, The University of Hong Kong, Hong Kong, China; 2 State Key Laboratory of Emerging Infectious Diseases, The University of Hong Kong, Hong Kong, China; 3 Research Centre of Infection and Immunology, The University of Hong Kong, Hong Kong, China; 4 Department of Biochemistry, The University of Hong Kong, Hong Kong, China; 5 Carol Yu Centre for Infection, The University of Hong Kong, Hong Kong, China

## Abstract

To elucidate the adaptive strategies of influenza A viruses (IAVs) to human, we proposed a computational approach to identify human-adaptive mutations in seasonal IAVs, which have not been analyzed comprehensively. We compared representative PB2 sequences of 1425 avian IAVs and 2176 human IAVs and identified a total of 42 human-adaptive markers, including 28 and 31 markers in PB2 proteins of seasonal viruses H1N1 and H3N2, respectively. Notably, this comprehensive list encompasses almost all the markers identified in prior computational studies and 21 novel markers including an experimentally verified mutation K526R, suggesting the predictive power of our method. The strength of our analysis derives from the enormous amount of recently available sequences as well as the recognition that human-adaptive mutations are not necessarily conserved across subtypes. We also utilized mutual information to profile the inter-residue coevolution in PB2 protein. A total of 35 and 46 coevolving site pairs are identified in H1N1 and H3N2, respectively. Interestingly, 13 out of the 28 (46.4%) identified markers in H1N1 and 16 out of the 31 (51.6%) in H3N2 are embraced in the coevolving pairs. Many of them are paired with well-characterized human-adaptive mutations, indicating potential epistatic effect of these coevolving residues in human adaptation. Additionally, we reconstructed the PB2 evolutionary history of seasonal IAVs and demonstrated the distinct adaptive pathway of PB2 segment after reassortment from H1 to H3 lineage. Our study may provide clues for further experimental validation of human-adaptive mutations and shed light on the human adaptation process of seasonal IAVs.

## Introduction

Influenza A virus (IAV) genome consists of 8 negative-sense RNA segments encoding 11–12 proteins^1^. The transcription and replication of influenza viruses are catalyzed by the viral polymerase complex composed of three subunits: polymerase basic protein 2 (PB2), polymerase basic protein 1 (PB1), and polymerase acidic protein (PA). The PB2 subunit binds the 5′ 7-methylguanosine cap of host pre-mRNAs, which are subsequently cleaved off 10–15 nucleotides downstream by PA^2,3^. PB1 protein, a viral RNA-dependent RNA polymerase, catalyzes the addition of nucleotides to the resulting capped short RNA primer and initiates viral transcription. The cooperation between the polymerase complex subunits is essential for viral replication and transcription^4^.

Migratory waterfowl is the natural reservoir of avian IAVs, from which IAVs are transmitted into other hosts, such as humans, domestic poultry, swine, and other species^5^. The host spectrum of influenza virus is mainly dictated by hemagglutinin (HA) glycoprotein and the viral polymerase complex^4^. Specific mutations in the receptor-binding domain of HA alter the specificity and affinity for the receptor and affect host tropism, while the mutations of viral polymerase proteins influence viral replication efficiency in new hosts^1,4^. Particularly, a single PB2 mutation E627K can cause highly increased replication efficiency and enhanced pathogenicity^6–8^, enabling the replication of avian-origin IAVs in human cells. In addition, the PB2 mutations D701N^9,10^, G590S/Q591R^11,12^, and K526R^13^ significantly increase replication efficiency in mammalian hosts.

Studies of human adaptation have been extended to other viral proteins. For example, T85I, G186S, and L336M in PA protein were identified to increase the polymerase activity of 2009 pandemic H1N1 (pH1N1) virus^14^. Amino acids L473V and L598P of PB1 protein from an avian-origin IAV contributed to higher polymerase activity, especially in mammalian cells^15^. Non-structural protein 1 mutations F103L and M106I led to increased viral growth of a human H5N1 isolate in vitro (mouse and canine cells) and enhanced virulence in mice^16^. Furthermore, epistatic effects of combinatorial mutations have been observed in IAV studies. Epistasis describes non-additive interactions among genetic sites, namely, the consequence of a mutation at one site depends on the presence of mutations at other sites. Epistasis commonly exists and plays an important role in immune escape and drug resistance in various pathogens^17^. In terms of the epistasis in IAVs, three PB2 mutations I147T, K399T, and A588T showed marginal effect when individually introduced into H5N1 but highly increased the polymerase activity when introduced in combination^18^.

To date, most of the human-adaptive mutations have been identified from epidemic or pandemic influenza virus isolates. However, those in seasonal human IAVs such as H1N1 and H3N2 subtypes remain poorly investigated. To fill the knowledge gap, we conducted a large-scale sequence analysis to identify the potential human-adaptive mutations in seasonal IAV PB2 protein and infer the adaptive evolutionary history of seasonal IAVs.

## Results

### Distribution of isolates and subtypes

On the basis that PB2 segments of seasonal H1N1 and H3N2 are both derivatives of 1918 pandemic H1N1 virus^1^, we aim to compare the PB2 segments of seasonal IAVs to avian IAVs for the discovery of potential adaptive markers in seasonal human IAVs. We surveyed 3457 and 6690 PB2 sequences of avian and human IAVs, respectively, with collecting years spanning from 1918 to 2016, in order to obtain a large pool of viral sequences. The distributions of collecting date and subtype were calculated. Avian IAVs are more diversified with 86 subtypes compared to 9 subtypes of human IAVs. The three major subtypes of avian viruses are H5N1 (16.7%), H3N8 (7.7%), and H6N2 (7.3%), while H1N1 (62.1%) and H3N2 (35.5%) are the dominant subtypes in human IAVs. We evaluated and eliminated sampling biases in both avian and human viruses, such as the oversampled H5N1 subtype in avian IAVs and the 2009 pandemic H1N1 in human IAVs. As a result, a total of 1425 avian and 2176 (1086 H1N1 and 1090 H3N2) human IAV sequences were retained for downstream analyses.

### Sites with host-specific amino acids in PB2

**Fig. 1:**
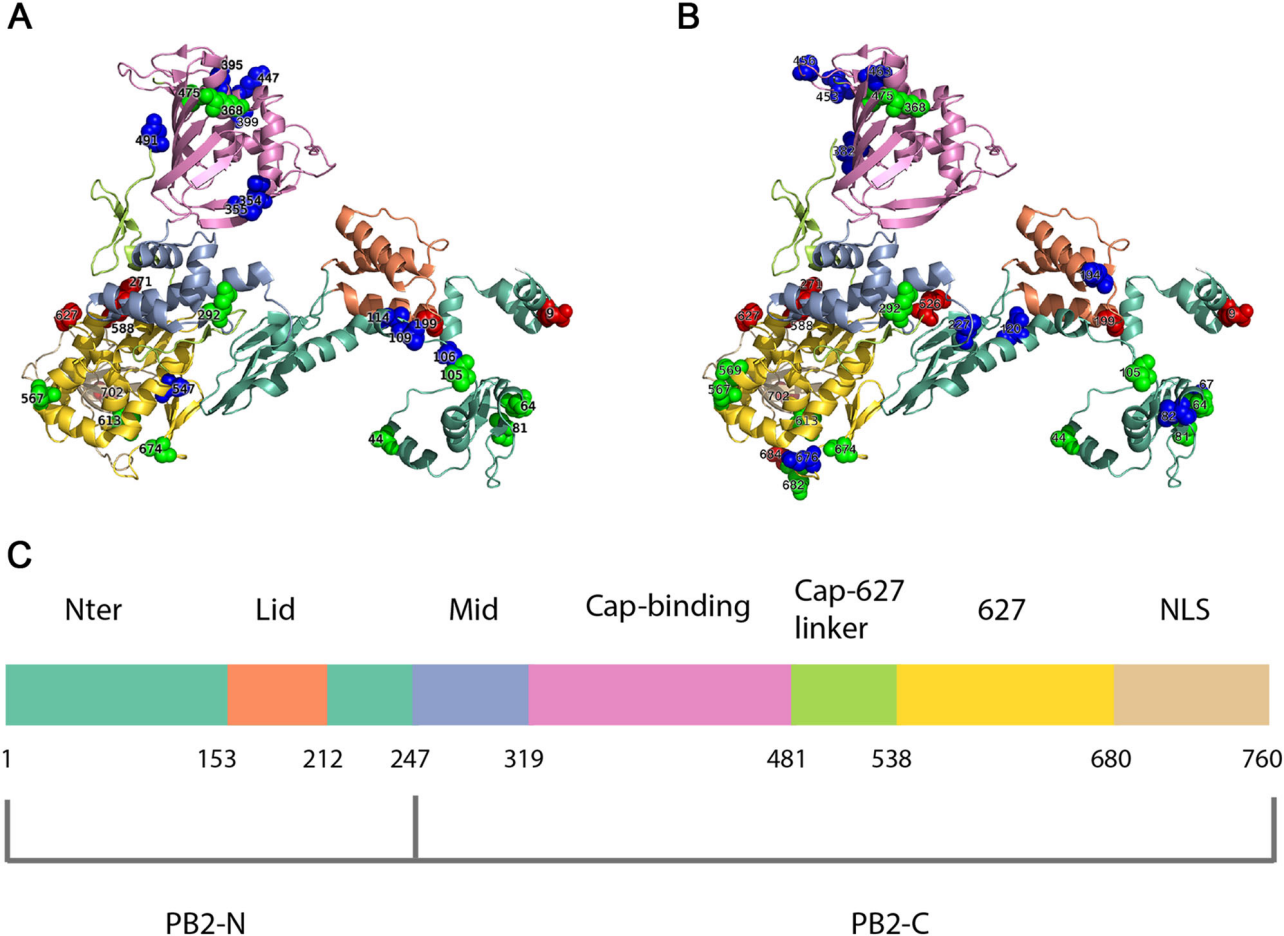
Host-specific signatures of seasonal IAVs in PB2 protein. Localization of identified human-adaptive markers in the PB2 domains of seasonal IAVs H1N1 (**a**) and H3N2 (**b**). The cartoon representation of PB2 subunit was rendered by Pymol (PDB code: 4WSB). Domains are colored according to the color code in **c**. Experimentally verified markers are indicated in red sphere, computationally predicted markers are indicated in green, and novel markers are indicated in blue. **c** The positions of PB2 domains are color-coded and labeled according to their functions

In order to identify human-adaptive markers in the PB2 protein, we compared the sequences of avian and seasonal human IAVs. Considering the genetic distinctions, seasonal H1N1 and H3N2 were compared to avian viruses separately. Using our comparative method, 28 and 31 human-adaptive markers in seasonal H1N1 (Supplementary Table S1) and H3N2 (Supplementary Table S2) were identified, respectively. Next, we asked whether any of the identified markers have been experimentally verified in previous studies, which may reflect the validity of our analysis. To this end, we compiled known experimentally validated human-adaptive mutations through an extensive literature review. As demonstrated in Table 1, to date, a total of 23 mutations in PB2 protein have been reported to increase replication efficiency and/or enhance pathogenicity significantly. We found that seasonal IAVs H1N1 and H3N2 harbour six and eight verified human-adaptive mutations, respectively. Among them, six mutations including D9N, A199S, T271A, A588I, E627K, and K702R are common in H1N1 and H3N2; while two mutations K526R and A684S are specific in H3N2, implicating that human-adaptive mutations are not necessarily conserved across subtypes as described in previous studies^19,20^. Additionally, we identified 12 markers, A44S, M64T, T81M, T105V, I292T, R368K, L475M, D567N, T569A, V613T, A674T, and G682S, which have been proposed to be human-adaptive markers in similar computational studies^19–22^. Notably, the other 11 markers, including T106A, V109I, V114I, I354L, R355T, A395V, I399V, Q447L, S490N, T491A, and V547I, in H1N1 and 10 markers, including I67V, N82S, E120D, Q194R, V227I, I382V, P453H, N456S, I463V, and T676I, in H3N2 have never been documented. A661T was excluded from both H1N1 and H3N2, since it was recently reported to have negligible effect on polymerase activity^23^. More specifically, among the 11 novel markers in H1N1, T106A, V109I, and V114I are located in Nter domain; I354L, R355T, A395V, I399V, and Q447L in cap-binding domain; S490N and T491A in cap-627 linker domain; and V547I in 627 domain. Comparatively, among the 10 novel markers in H3N2, I67V, N82S, E120D, and V227I are located in Nter domain; Q194R in Lid domain; I382V, P453H, N456S, and I463V in cap-binding domain; and T676I in 627 domain (Fig. 1). Taken together, by comparing the amino acid distribution of PB2 protein between avian and human IAVs, we identified 28 and 31 human-adaptive markers in H1N1 and H3N2 subtypes, respectively. More than half of them have been either experimentally verified or repeatedly predicted in previous computational studies. Additionally, we also pinpointed novel markers of human adaptation that reside in well-defined functional domains of PB2 protein.

**Table 1:** List of experimentally verified PB2 human-adaptive mutations

AA substitution^a^	Identified subtype(s)	Phenotypes	References
D9N	H1N1	Increased polymerase activity, enhanced pathogenicity in mice	^28, 42^
M147T/L	H5N1	Increased polymerase activity	^18, 43^
E158G	H1N1 and H5N1	Increased polymerase activity, enhanced pathogenicity in mice	^44, 45^
A199S	H5N1	Increased polymerase activity	^28^
E249G	H5N1	Increased polymerase activity	^46^
D253N	H9N2	Increased polymerase activity, enhanced pathogenicity in mice	^47^
D256G	H5N1	Increased polymerase activity	^48^
T271A	pH1N1 and H5N1	Increased polymerase activity	^12, 49, 50^
G309D	H5N1	Increased polymerase activity	^46^
K339T/M	H5N1	Increased polymerase activity	^18, 46^
K526R	H5N1	Increased polymerase activity, enhanced pathogenicity in mice	^13^
M535T/L	H5N1	Increased polymerase activity	^51, 52^
A588I/V	pH1N1, H7N9, H9N2, and H10N8	Increased polymerase activity, enhanced pathogenicity in mice	^53, 54^
G590S/Q591R	pH1N1, H9N2	Increased polymerase activity, enhanced pathogenicity in mice	^11, 12, 47^
E627K	H1N1, H5N1, and H7N9	Increased polymerase activity, enhanced pathogenicity in mice	^6, 10^
L636F	H1N1	Increased polymerase activity	^55^
V661A	pH1N1	Increased polymerase activity at low temperature	^50^
V683T/A684S	pH1N1	Increased polymerase activity at low temperature	^50^
D701N	H5N1	Increased polymerase activity, enhanced pathogenicity in mice	^9, 10^
K702R	H5N1	Increased polymerase activity, enhanced pathogenicity in mice	^52^
S714R	H5N1	Increased polymerase activity, enhanced pathogenicity in mice	^55^

^a^H5 numbering system was used to represent amino acid substitutions

### Sites of coevolution in PB2

**Fig. 2:**
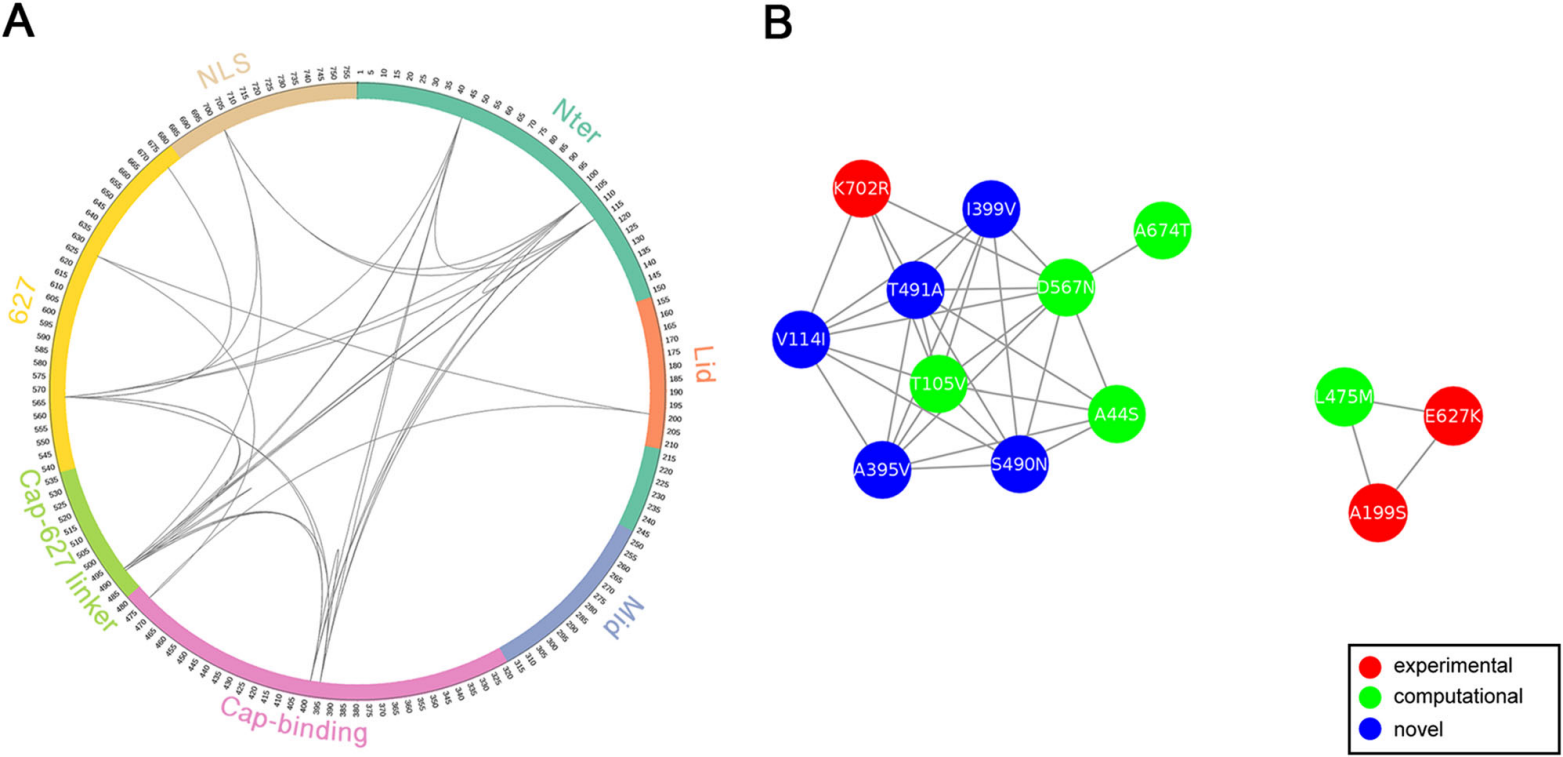
MI network of seasonal H1N1 IAV PB2 protein. **a** Circular representation of PB2 protein and the MI network. The positions of PB2 domains are color-coded and labeled according to their functions. Arcs connect site pairs with normalized MI > 0.7. **b** Cytoscape representation of the MI network. Sites are represented as nodes. Edge between two nodes indicates the normalized MI > 0.7. Sites with experimentally verified human-adaptive mutations and computationally identified markers are highlighted in red and green, respectively, in **a**, **b**; blue nodes indicate novel markers

**Fig. 3:**
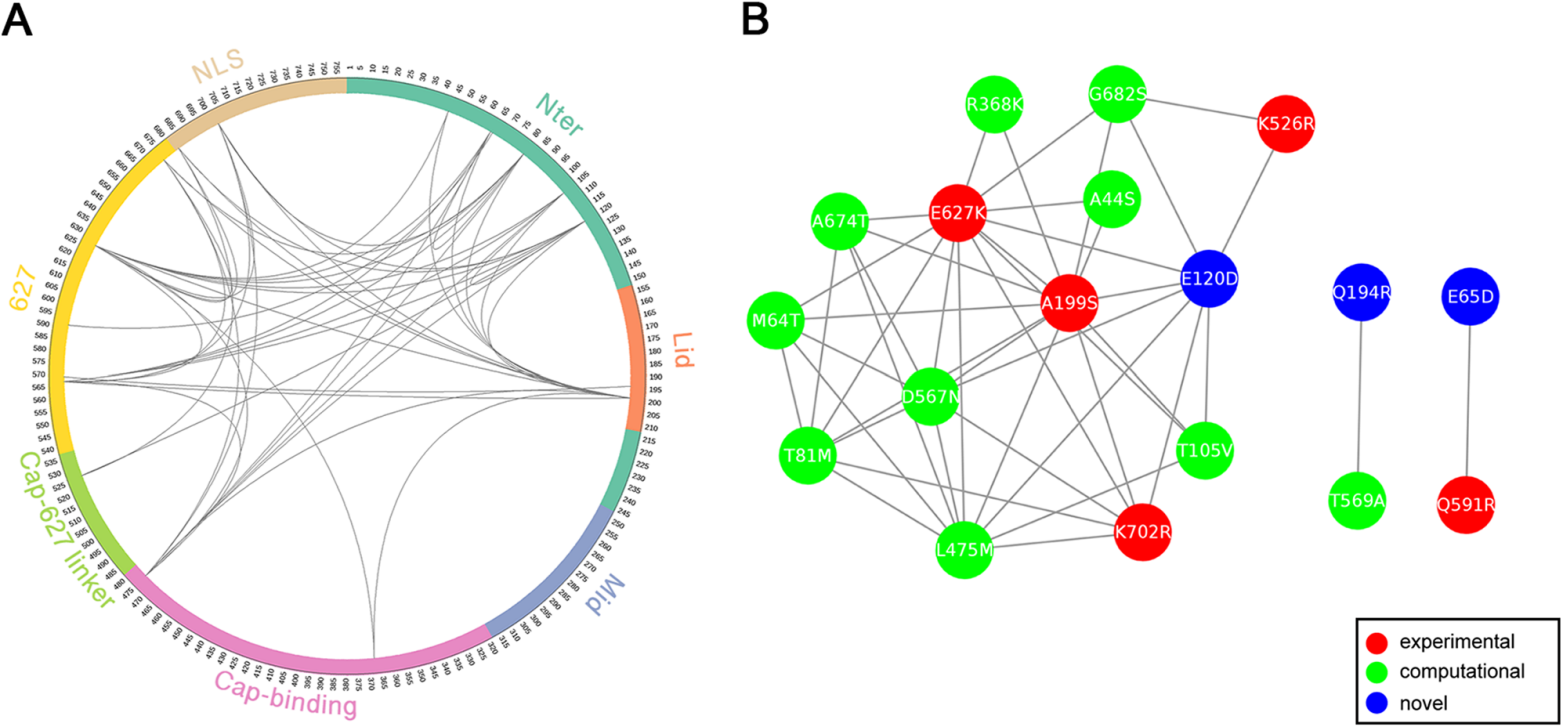
MI network of seasonal H3N2 IAV PB2 protein. **a** Circular representation of PB2 protein and the MI network. The positions of PB2 domains are color-coded and labeled according to their functions. Arcs connect site pairs with normalized MI > 0.7. **b** Cytoscape representation of the MI network. Sites are represented as nodes. Edge between two nodes indicates the normalized MI > 0.7. Sites with experimentally verified human-adaptive mutations and computationally identified markers are highlighted in red and green, respectively, in **a**, **b**; blue nodes indicate novel markers

Patterns of amino acid conservation across a large set of homologs can be utilized to identify structurally or functionally important residues; meanwhile, patterns of correlated substitutions or amino acid covariation can also reveal important residues^24^. To explore the coevolution profile of PB2 protein in H1N1 and H3N2 viruses, we quantified the covariation between site pairs with mutual information (MI)^25^. Since identification of coevolution with MI requires sufficiently large alignment of homologous sequences^24^, we combined avian, swine, and seasonal human IAV PB2 sequences. However, MI values can be misleading when homologous sequences are not collected properly or the sequence alignment is not built correctly^26^. In order to take different homologous sequences into account more equivalently and to make the MI values more comparable between H1N1 and H3N2, we performed resampling to construct balanced samples with equal number of avian, swine, and seasonal human IAVs. Using this approach, we identified 35 (Supplementary Table S3) and 46 (Supplementary Table S4) coevolving site pairs, embracing 13 (Fig. 2) and 18 (Fig. 3) identified markers in PB2 of H1N1 and H3N2, respectively. These coevolving sites are distributed in almost all domains except Mid domain (Figs. 2a and 3a). The Nter, cap-binding, and 627 domains are highly connected with each other in both H1N1 and H3N2 coevolution networks, consistent to the idea that the N-terminal third of PB2 (amino acids 1–247) is not only structurally but also functionally a part of the polymerase core^27^. Notably, the epistatic effects of a number of coevolving pairs have been demonstrated in previous wet-lab studies. For instance, sites 199 and 627 show high covariation with each other in both H1N1 and H3N2 subtypes. Consistently, the significant synergistic effect of A199S and E627K on viral replication and pathogenicity was observed in H5N1 subtype^28^, which can lend support to the validity of our approach.

We also noted that multiple verified human-adaptive mutations are present in the coevolution networks, in connection with other identified markers. As shown in H1N1 coevolution network (Fig. 2b), the well-characterized human-adaptive mutations A199S and E627K are connected with a computational marker L475M. Another verified human-adaptive mutation K702R is connected with two computational markers and two novel markers identified in our study. The more extensive connections in H3N2 coevolution network are demonstrated in Fig. 3. The four well-characterized mutations A199S, K526R, E627K, and K702R are connected with nine computational markers and a novel marker E120D. The coevolution between site pairs indicates their structural or functional importance in conformation stabilization of the polymerase or in adaptation into different hosts. Collectively, we disclosed extensive coevolution networks in PB2 protein of H1N1 and H3N2 subtypes and demonstrated that a large portion (>40%) of the identified human-adaptive markers exhibit significant coevolution.

### Adaptive evolutionary history of PB2 protein

**Fig. 4:**
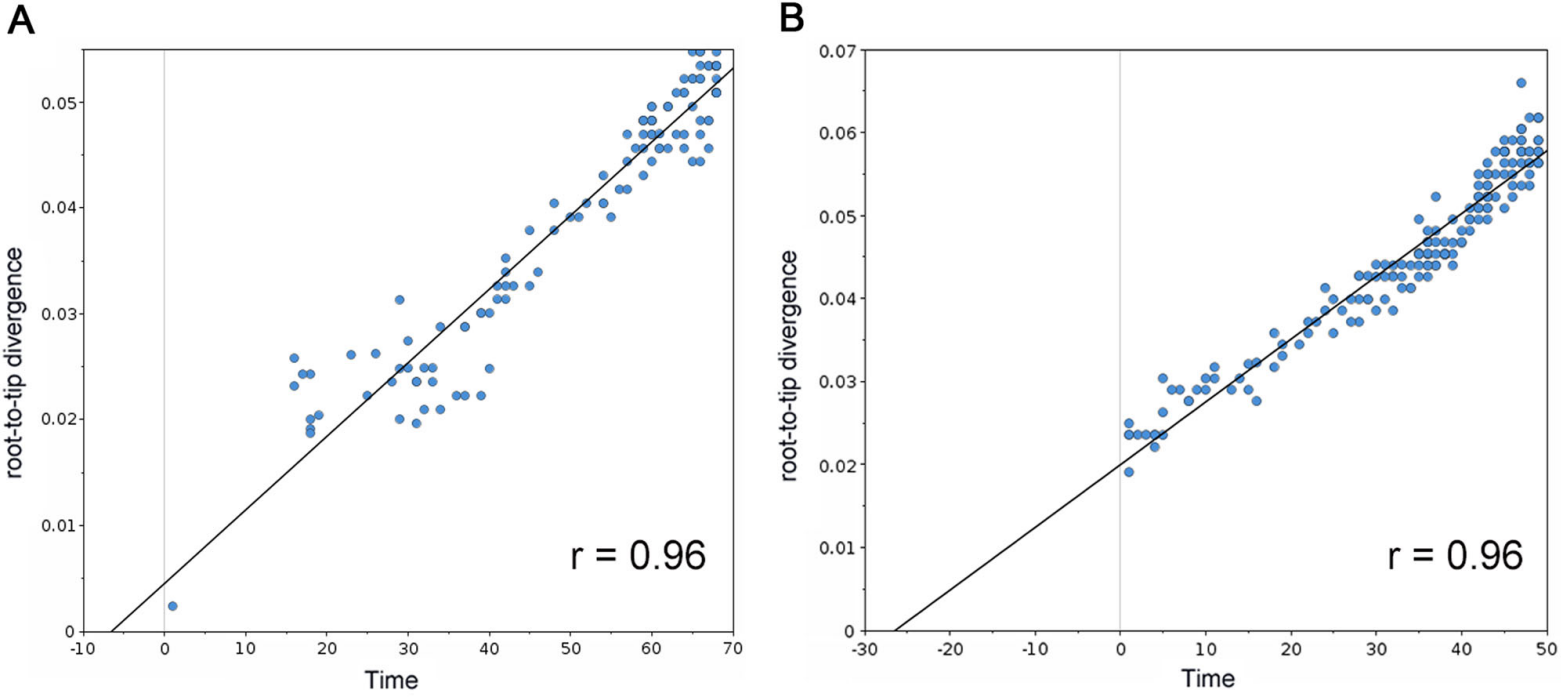
Correlation of root-to-tip divergence with evolution time. **a** Correlation of representative H1N1 PB2 sequences from 1918 to 2009 with root-to-tip divergence. **b** Correlation of representative H3N2 PB2 sequences from 1968 to 2016 with root-to-tip divergence. The *x* axis represents the evolution time of the MRCA; the *y* axis represents the sequence divergence from the MRCA. Each dot corresponds to a PB2 protein sequence

In an effort to understand how the identified markers emerged temporally, we reconstructed the most recent common ancestor (MRCA) (Supplementary Text S1) and the evolutionary history of PB2 segments of seasonal IAVs with Bayesian phylogenetic inference. The representative sequences of evolutionary history were selected with the linear regression of root-to-tip genetic distance against divergence time under the strict molecular clock. As shown in Fig. 4, 115 (Supplementary Text S2) and 170 (Supplementary Text S3) sequences are retained for the reconstruction of evolutionary history of H1N1 and H3N2 respectively, with regression *r*^2^ exceeding 0.9.

**Fig. 5:**
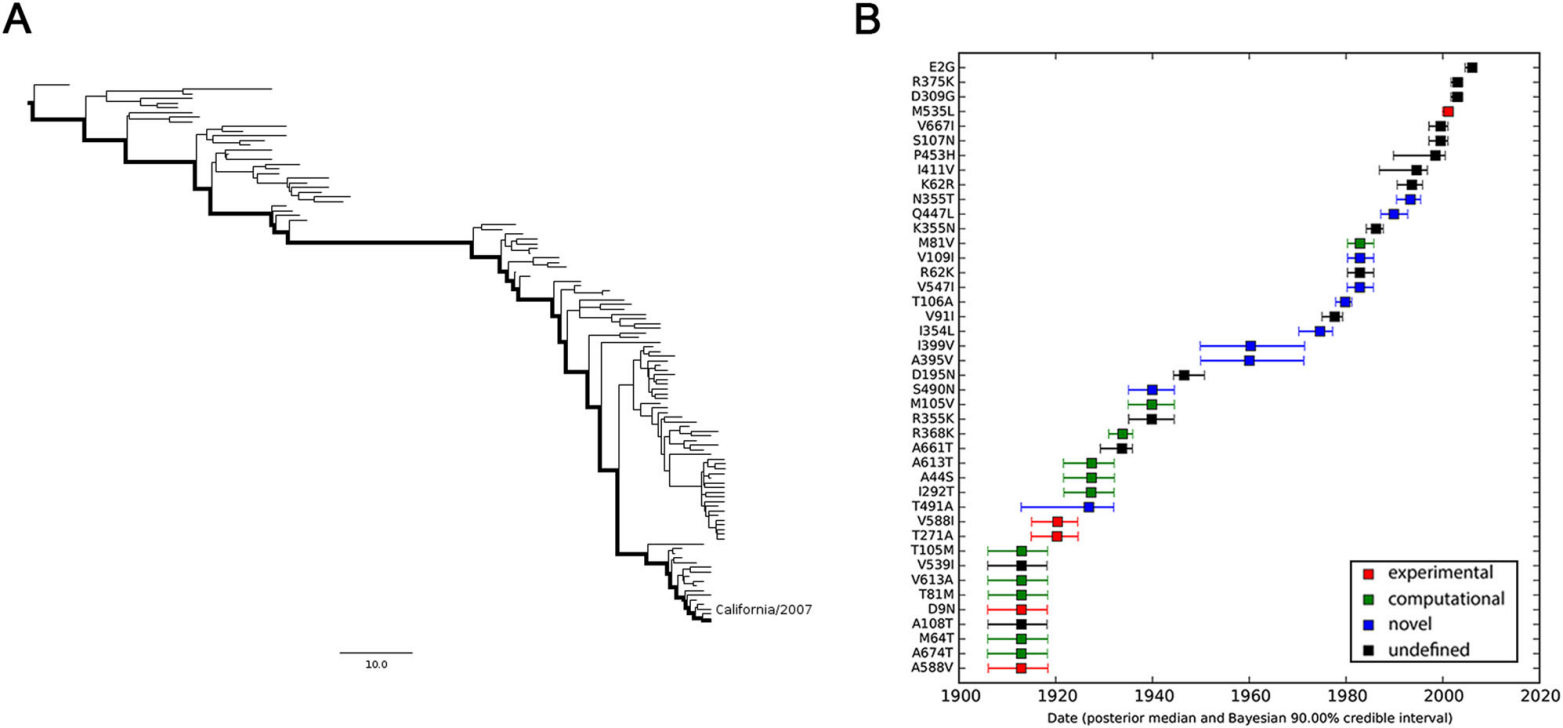
MCC tree and mutational path of seasonal H1N1 IAV. **a** MCC tree of seasonal H1N1 IAVs circulated between 1918 and 2009. A total of 115 representative PB2 sequences were selected to reconstruct the tree by Bayesian phylogenetic inference. The evolutionary path from the MRCA to the most divergent descendant (A/California/6/2007) is highlighted in the tree. **b** Reconstructed mutational path from the MRCA to the most divergent descendant. The *x* and *y* axes represent the time scale and the mutations in the path, respectively. The median and 90% BCI of estimated date of each mutation are shown in the boxplot. Experimentally verified markers, computationally predicted markers, and novel markers are indicated in red, green, and blue, respectively

**Fig. 6:**
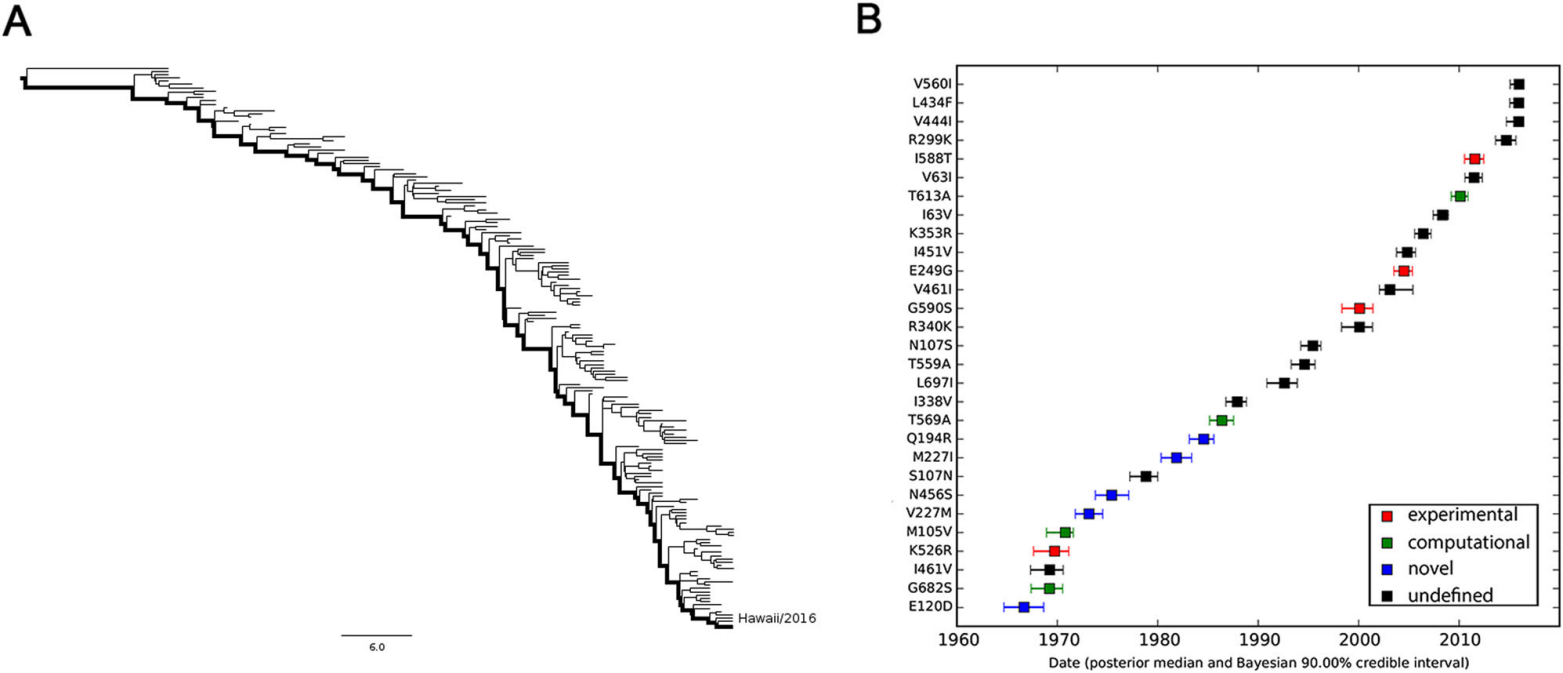
MCC tree and mutational path of seasonal H3N2 IAV. **a** MCC tree of seasonal H3N2 IAVs circulating between 1968 and 2016. A total of 170 representative PB2 sequences were selected to reconstruct the tree by Bayesian phylogenetic inference. The evolutionary path from the MRCA to the most divergent descendant (A/Hawaii/13/2016) is highlighted in the tree. **b** Reconstructed mutational path from the MRCA to the most divergent descendant. The *x* and *y* axes represent the time scale and the mutations in the path, respectively. The median and 90% BCI of estimated date of each mutation are shown in the boxplot. Experimentally verified markers, computationally predicted markers, and novel markers are indicated in red, green, and blue, respectively

The maximum clade credibility (MCC) tree and the corresponding mutational path were summarized from the Bayesian Markov chain Monte Carlo (MCMC) simulations. The MCC trees illustrating the evolution of the PB2 gene of seasonal H1N1 and H3N2 have a “cactus-like” shape with a strong temporal structure (Figs. 5a and 6a). The trunk represents the succession of surviving viral lineages over time while short side branches indicate the extinction of most strains. The evolutionary history of seasonal H1N1 from 1918 to 2009 (replaced by the 2009 pH1N1^29,30^) was reconstructed with 115 sequences, with roughly 1.26 sequences per year. In contrast, the evolutionary history of H3N2 from 1968 to 2016 is covered by 3.54 sequences per year. Accordingly, the mutational path of H3N2 (Fig. 6b) is more definite than that of H1N1 (Fig. 5b).

We also estimated the emerging dates of the identified markers (Table 2). Among the 28 identified human-adaptive markers in H1N1, the MRCA of H1N1 PB2 harbored 6 markers originally and acquired the other 22 markers until 1993 (90% Bayesian credible interval, BCI, 1990–1995). Interestingly, three out of the six markers, A199S, E627K, and K702R, are top-ranked human-adaptive mutations, suggesting their importance in crossing the species barrier at the early stage. The remaining three verified human-adaptive mutations D9N, T271A, and A588I were acquired before 1922 (BCI 1917–1926). In contrast, among the 31 identified markers in H3N2 IAVs, the MRCA of H3N2 PB2 harbored 23 markers originally, of which 7 markers have been experimentally verified (Table 2). The remaining 8 markers were acquired sequentially within around 25 years, the last verified human-adaptive mutation K526R was acquired in 1969 (BCI 1967–1971). Therefore, the PB2 segment of H3N2 developed a distinct adaptive pathway compared to H1N1. The PB2 protein of seasonal H1N1 viruses may originate from a limitedly adapted avian-origin IAV. It acquired most of the human-adaptive mutations during circulating in human population. On the contrary, the PB2 segment of seasonal H3N2 was derived from a well-adapted predecessor originating from seasonal H1N1 viruses through segment reassortment^1^. It acquired human adaptation mutations, such as K526R and A684S, which are absent in seasonal H1N1 viruses.

**Table 2:** Estimated emerging dates of identified markers in seasonal H1N1 and H3N2 PB2

H1N1			H3N2		
Site	Residue	Date	Site	Residue	Date
114	I	Pre-existed	9^a^	N	Pre-existed
199^a^	S	Pre-existed	44	S	Pre-existed
475	M	Pre-existed	64	T	Pre-existed
567	N	Pre-existed	67	V	Pre-existed
627^a^	K	Pre-existed	81	M	Pre-existed
702^a^	R	Pre-existed	82	S	Pre-existed
674	T	1914.87 (BCI 1907.89–1920.29)	199^a^	S	Pre-existed
64	T	1914.89 (BCI 1907.94–1920.36)	271^a^	A	Pre-existed
9^a^	N	1914.91 (BCI 1907.93–1920.25)	292	T	Pre-existed
271^a^	A	1922.26 (BCI 1916.92–1926.62)	382	V	Pre-existed
588^a^	I	1922.38 (BCI 1917.00–1926.55)	453	H	Pre-existed
491	A	1928.86 (BCI 1914.84–1933.99)	463	V	Pre-existed
292	T	1929.32 (BCI 1923.67–1934.04)	475	M	Pre-existed
44	S	1929.40 (BCI 1923.61–1934.12)	567	N	Pre-existed
613	T	1929.43 (BCI 1923.59–1934.09)	588^a^	I	Pre-existed
368	K	1935.82 (BCI 1932.95–1937.92)	613	T	Pre-existed
105	V	1941.87 (BCI 1936.96–1946.51)	627^a^	K	Pre-existed
490	N	1941.96 (BCI 1937.03–1946.51)	674	T	Pre-existed
395	V	1960.42 (BCI 1950.35–1971.67)	676	I	Pre-existed
399	V	1960.69 (BCI 1950.27–1971.84)	684^a^	S	Pre-existed
354	L	1975.02 (BCI 1970.64–1977.62)	702^a^	R	Pre-existed
106	A	1980.24 (BCI 1978.24–1981.58)	368	K	Pre-existed
547	I	1983.25 (BCI 1980.63–1986.06)	120	D	1966.70 (BCI 1964.70–1968.65)
109	I	1983.30 (BCI 1980.72–1986.13)	682	S	1969.23 (BCI 1967.39–1970.53)
81	V	1983.30 (BCI 1980.70–1986.15)	526^a^	R	1969.73 (BCI 1967.63–1971.13)
447	L	1990.29 (BCI 1987.59–1993.15)	105	V	1970.79 (BCI 1968.93–1971.59)
355	T	1993.71 (BCI 1990.84–1995.82)	227	I	1975.41 (BCI 1973.80–1977.11)
			456	S	1981.87 (BCI 1980.32–1983.38)
			194	R	1984.58 (BCI 1983.14–1985.58)
			569	A	1986.39 (BCI 1985.15–1987.56)

^a^Experimentally verified human-adaptive markers

## Discussion

Adaptive mutations in the PB2 polymerase play a critical role for avian influenza virus replication in mammalian cells and enable some avian IAVs to establish infection in humans. In this study, we conducted a large-scale sequence comparison between avian and human IAVs, whereby a comprehensive list of novel human-adaptive markers in PB2 protein of H1N1 and H3N2 viruses were identified. To our knowledge, this is basically an exhaustive list, which encompasses most well-characterized human-adaptive markers in PB2 protein identified in prior studies as well as the novel markers obtained from this study. The identification of a large pool of adaptive markers allows us to uncover the coevolution pattern among these markers, which implicates their correlated function in host adaptation. Additionally, we demonstrated the distinct evolutionary pathways of seasonal H1N1 and H3N2 viruses.

Previous similar computational studies tend to focus on human-adaptive mutations that are conserved in all subtypes^19^. Intriguingly, we noted that two well-characterized human-adaptive mutations K526R and A684S are specific in the H3N2 subtype, suggesting the necessary concerns for subtype-specific mutations. Therefore, we relaxed the assumption and compared seasonal H1N1 and H3N2 to avian viruses separately. With our approach, a number of novel markers that have not been reported in similar computational studies were identified in both H1N1 and H3N2 subtypes. Notably, an experimentally verified mutation K526R, which has not been identified in previous computational studies^19,20,31^, was revealed in our study. The outperformance of our approach may originate from two improvements. First, we relaxed the assumption in previous approaches that human-adaptive mutations should be conserved across subtypes as aforementioned. Second, the dramatically expanding databases provide large-scale thoroughly sampled sequence data. With the availability of comprehensive sequence data of seasonal IAVs, we can significantly improve the predictive power.

There has been a growing recognition that epistasis plays a key role in functional evolution of proteins by constraining accessible evolutionary pathways and increasing the role of contingency in adaptation^17,32,33^. The functional effect of a given substitution frequently depends on the presence or absence of other substitution(s)^17^. Thus epistatic sites usually present covariation or coevolution and exhibit particular patterns in multiple sequence alignment (MSA) of homologous proteins. The algorithms for detecting coevolution can be divided into two general classes: algorithms such as MI and statistical coupling analysis that score covariation between all pairs of columns in a sequence alignment; while global probabilistic models such as direct coupling analysis that assess the likelihood of covariation between sites^24^. The former algorithm has been utilized in our study to quantify the inter-residue covariation, since it is conceptually straightforward, technically simpler to implement, and often sufficiently powerful to provide useful insights of coevolution.

Analyses of amino acid coevolution within protein family can serve as a valuable guide for identifying residues that are functionally coupled^24^. Indeed, a number of the identified coevolving pairs in this study showed significant epistasis in previous wet-lab studies. For instance, combination of coevolving pair A199S and E627K imposed a strong synergistic effect on replication efficiency^28^. R368K, a coevolving partner of E627K, showed limited effect when introduced alone into a H5N1 virus strain but significantly increased replication efficiency and pathogenicity when combined with 627K^28^. Moreover, the adaptive effects of a number of mutations in PA and NP proteins also showed dependency on E627K. For instance, three NP mutations R100V/I and L283P^28^ can cause a failure of virus rescue but showed highly enhanced replication efficiency with 627K^28^. Integrating the prior findings and discoveries obtained from our study, we believe that the strong human-adaptive effect may not solely result from the verified mutations as demonstrated previously^6^. Instead, the effects of verified mutations may depend on the intricate interplays with other mutations. Currently, we only focused on the inter-residue coevolution within PB2 protein due to the lack of adequate whole-genome sequences. Upon the availability of sufficient whole-genome sequences in the future, inter-protein coevolution analysis could uncover important residues that are involved in protein–protein interactions.

This study, like similar studies, is sensitive to sampling biases that have to be estimated and controlled properly. Otherwise, the true distinctions would probably be masked. For instance, H1N1 sequences were highly oversampled in 2009 during the pandemic so that the number of pH1N1 sequences is far more than the total amount of seasonal H1N1 in public databases. Additionally, pH1N1 actually derived from multiple reassortment events and contains a swine-origin PB2 segment^34,35^, which may override the true distinctions between avian and seasonal human IAVs. Therefore, we carefully eliminated pH1N1 prior to comparison. Despite all the efforts, our comparative method does have limitations. We are unable to identify newly fixed adaptive mutations, since they are unlikely to achieve predominance to fulfill the first criterion. For instance, two verified mutations M535L and E249G, which were acquired by seasonal H1N1 (Fig. 5b) and H3N2 (Fig. 6b) subtypes recently, are not identified with our method.

In summary, we designed a simple approach to study the human adaptation of seasonal IAVs. We identified a large number of human-adaptive markers and profiled the coevolution among them. In addition, we inferred the MRCA and adaptive evolutionary history of seasonal IAVs and estimated the emerging dates and sequential order of each identified markers. We believe that our findings will provide clues for further experimental validation of singular and combinatorial human-adaptive mutations and shed light on the human adaptation process of seasonal IAVs.

## Materials and methods

### Sequence preprocessing

The PB2 sequences of avian and human IAVs were retrieved from the OpenFluDB database (http://openflu.vital-it.ch). Subtype distributions of both avian and human IAVs were estimated. For avian IAV viruses, replicate sequences within the same subtype due to oversampling were removed. For human IAV viruses, non-seasonal subtypes such as 2009 pandemic H1N1 (3083 sequences), H5N1 (367 sequences), and H7N9 were excluded. The remaining IAVs were subdivided into two subsets, H1N1 and H3N2. Replicate sequences within each subset collected in the same year were excluded to eliminate oversampling in certain years. Nonsense characters of each sequence were trimmed while those with partial length (<759 aa) were removed. MSA of PB2 was constructed using the Muscle v3.7^36^ software with the fastest parameters (-maxiters 2 without refinement), due to the large amount of sequences.

### Sites with host-specific amino acids

Site-wise amino acid compositions in PB2 of H1N1 and H3N2 subsets were compared to those of avian IAVs, respectively. Frequencies (*F*) of amino acids in each aligned position were calculated. The predominant amino acid of each site is defined as that with the largest *F*. Pearson’s chi-square test was used to test the statistical significance of amino acid distribution difference in the corresponding position between avian and human IAVs. Cramer’s *V* test was utilized to normalize the chi-square statistic to control for dataset size and quantify the effect size. Accordingly, two criteria were employed to define an amino acid as a human-adaptive marker in a given site: (i) the frequency of the amino acid is predominant (*F* > 0.5) in human IAVs and minor (*F* < 0.5) in avian IAVs; (ii) the Cramer’s *V* value is >0.8. The marker is indicated as “*A* + site + *B*” where *A* and *B* are the predominant amino acids of avian and human IAVs, respectively, in that site and the substitution from *A* to *B* is assumed to be responsible for human adaptation. Only the counts of amino acid *A* and *B* in each aligned position of avian and human IAVs were taken into tests to ensure the same degree. We used 1% of the total count as a pseudo value in tests when the observed count is <5. The sequence comparison was implemented using Python and Biopython framework^37^. Statistical tests were performed using R language^38^. Sites with host-specific amino acids were mapped onto the polymerase complex (PDB code: 4WSB) by using Pymol. The source code is available upon request.

### Coevolution analysis

Shannon’s entropy (*H*) is a measure of uncertainty or randomness^39^. The entropy of a column *c* in a MSA is calculated with the following equation:

$$H_c = - \mathop {\sum }\limits_{i = 1}^{20} p(x_i)log_{20}p(x_i)$$

Here *p(x*_*i*_*)* is the observed frequency of amino acid *i* occurring at a site. All values were calculated using a log_20_ so that the range of position entropy scores is 0–1. The covariation between two sites is quantified using MI, which quantifies the mutual dependence between two variables. The MI between two positions in a MSA is given as

$${\mathrm{MI}}\left( {c,d} \right) = H_c + H_d - H_{cd}$$

where *H*_*c*_ and *H*_*d*_ are entropies of column *c* and *d*, respectively, and *H*_*cd*_ is the joint entropy of column *c* and *d* calculated by the same method using the frequencies of occurrence of each combination of residues in column *c* and *d*. The MI scores range from 0 to the minimum of *H*_*c*_ or *H*_*d*_. The raw MI values are normalized by dividing by the joint entropy of the positions, *H*_*cd*_, to reduce the influence of entropy on MI. Normalized MI values range from 0 to 1^25^.

Homologous sequence alignment for covariation quantification was constructed with avian, swine, and human IAV PB2 sequences of the same subtype. All sequences were retrieved from OpenFluDB. Replicates in each virus subset were excluded for computational simplicity. Owing to the sensitivity of MI to sampling balance, re-sampling was performed for 1000 times, in which we randomly extracted equal number of sequences from each host category with replacement to construct balanced samples. The number of extraction was determined by the minimum sequence count of the subsets. Average MI values between all site pairs of the 1000 reconstructed samples were then calculated. Site pairs with average MI value >0.7 and average entropy values of both sites >0.2 were designated as coevolving sites. Entropy and MI value calculation was implemented in custom python scripts.

### MCC tree and evolutionary history reconstruction

Preprocessed human seasonal IAV sequences were used for the construction of MCC tree and evolutionary history. A maximum of 10 sequences of H1N1 and H3N2 per year were retained to avoid the trees being too large. Next, RAxML^40^ (version 8.2.9; http://sco.hits.org/exelixis/web/soft-ware/raxml/) was utilized to infer the parsimony tree with JTT model. The tree was then visually analyzed using TempEst^41^ (version 1.5; http://tree.bio.ed.ac.uk/software/tempest/) to identify potential violations that substantially deviated from the linear regression of root-to-tip genetic distance against divergence time. We removed the outliers and then repeated these two steps to achieve a high consistence between molecular clock and stamped dates. The remaining sequences were used in downstream analyses.

To infer the evolutionary history and the MRCA for the PB2 sequences, a Bayesian MCMC method was applied as implemented in the BEAST package (version 1.8.3; http://beast.bio.ed.ac.uk). BEAGLE (http://beast.bio.ed.ac.uk/BEAGLE) was utilized to boost the core computation. The BEAST XML input file was generated using the combination of BEAUTI (inside BEAST package) and hand-annotation. This XML file that specifies date-stamped sequences, a strict molecular clock and a JTT model of substitution, was used in multiple runs of MCMC simulation. The MCMC chain was set as 100 million iterations, with subsampling every 10,000 iterations. Tracer (version 1.6; http://beast.bio.ed.ac.uk/tracer) was used to track the log file of combined runs with the initial 10% of the chain as burn-in to ensure good MCMC convergence. The MCC tree was summarized using TreeAnnotator (version 1.8.3; inside BEAST package) on the basis of merged simulations. FigTree (version 1.4.2; http://tree.bio.ed.ac.uk/software/figtree) was used to visualize the tree file, manually recolor the branches, and export to tree images. The mutational path was extracted from the merged trees using Mutpath python package^33^ (available at http://github.com/jbloom/mutpath), with manually created input file.

### Availability of data and material

The datasets used and/or analyzed during the current study are available from the corresponding author on request.

## Electronic supplementary material

Figure S1

Table S1

Table S2

Table S3

Table S4

Text S1

Text S2

Text S3

Supplementary Figure an Table legend
