# Supplementary figures and images for: Large-scale sequence analysis reveals novel human-adaptive markers in PB2 segment of seasonal influenza A viruses
Source: Emerg Microbes Infect. 2018 Mar 29;7:47. doi: 10.1038/s41426-018-0050-0 (PMC5874250; doi:10.1038/s41426-018-0050-0)

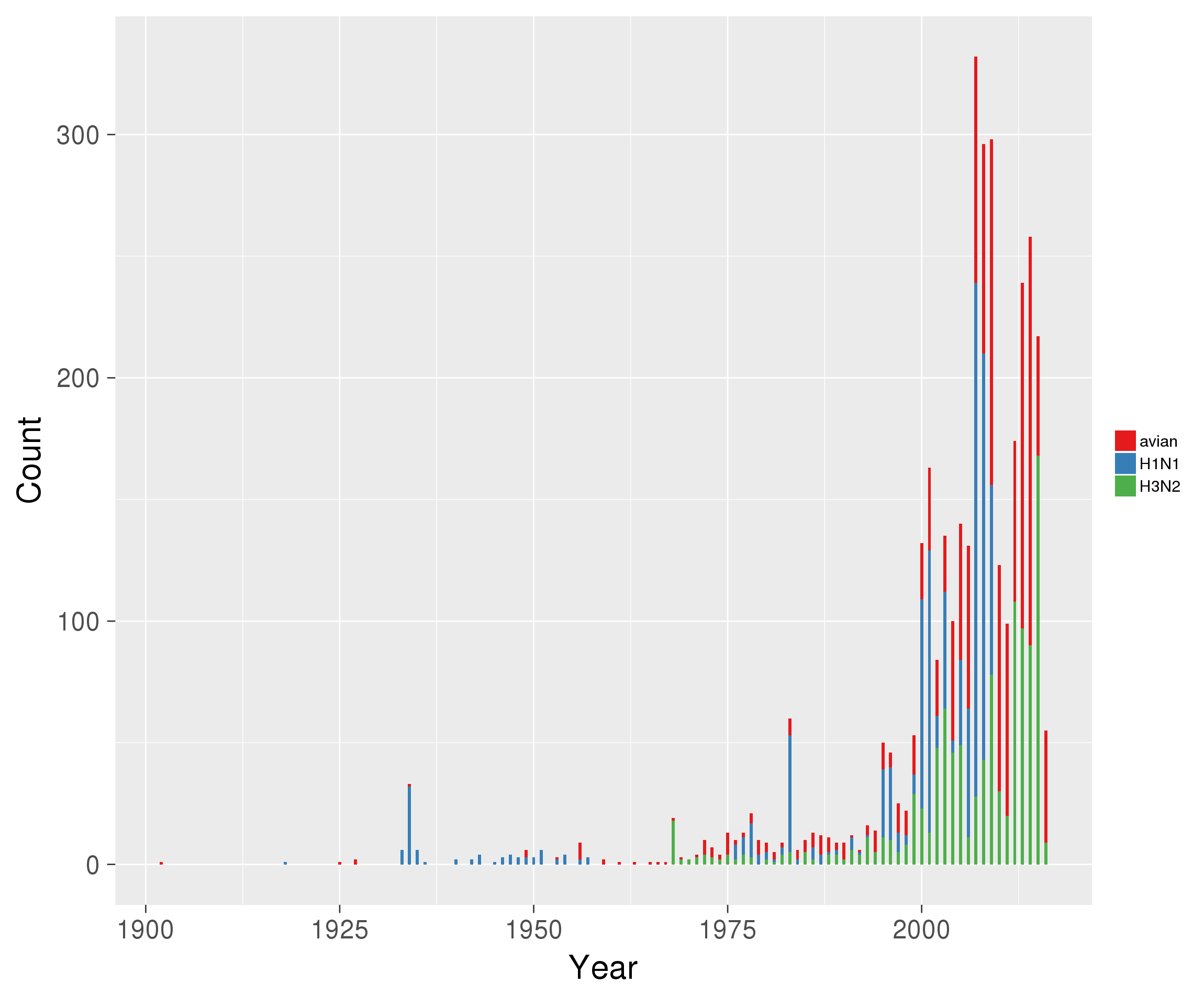

Supplement: Supplementary file 1 — Figure S1 [file 41426_2018_50_MOESM1_ESM.tif]
