# Supplementary material for: Large-scale sequence analysis reveals novel human-adaptive markers in PB2 segment of seasonal influenza A viruses
Source: Emerg Microbes Infect. 2018 Mar 29;7:47. doi: 10.1038/s41426-018-0050-0 (PMC5874250; doi:10.1038/s41426-018-0050-0)
Supplement: Supplementary file 9 — Supplementary Figure an Table legend [file 41426_2018_50_MOESM9_ESM.docx]

**Supplementary**

**Figure S1.**

The numbers of retained avian, seasonal H1N1 and H3N2 sequences in each year from 1918 to 2016 after preprocessing.

**Text S1.**

The inferred sequences of the most recent common ancestor (MRCA) of H1N1 and H3N2 PB2 proteins.

**Text S2.**

The 115 representative PB2 sequences of H1N1 used in Markov chain Monte Carlo (MCMC) simulations and Bayesian phylogenetic analysis.

**Text S3.**

The 170 representative PB2 sequences of H3N2 used in Markov chain Monte Carlo (MCMC) simulations and Bayesian phylogenetic analysis.

**Table S1.**

Full catalogue of identified human-adaptive markers in IAV H1N1 PB2 protein. Each row represents a site with the columns detailing the sites of human-adaptive marker, amino acid distribution of the site in avian IAVs, amino acid distribution of the site in human IAVs and Cramer's V value.

**Table S2.**

Full catalogue of identified human-adaptive markers in IAV H3N2 PB2 protein. Each row represents a site with the columns detailing the sites of human-adaptive marker, amino acid distribution of the site in avian IAVs, amino acid distribution of the site in human IAVs and Cramer's V value.

**Table S3.**

List of putative coevolution pairs with mutual information (MI) value > 0.7 identified in H1N1 PB2 protein.

**Table S4.**

List of putative coevolution pairs with mutual information (MI) value > 0.7 identified in H3N2 PB2 protein.
